# Sex-Specific Differences in Sepsis Development in Polytrauma Patients Undergoing Stand-Alone Definitive Surgery

**DOI:** 10.3390/medicina61020183

**Published:** 2025-01-22

**Authors:** Philipp Vetter, Cédric Niggli, Jan Hambrecht, Hans-Christoph Pape, Ladislav Mica

**Affiliations:** Department of Trauma Surgery, University Hospital Zurich, 8091 Zurich, Switzerland

**Keywords:** polytrauma, sex, definitive surgery, sepsis

## Abstract

*Background and Objectives:* In the triaging of polytrauma, patients with less severe injuries and lower somatic stress often undergo isolated definitive orthopedic surgery without damage-control procedures. Adverse events, particularly sepsis, should be minimized. We aimed to identify sex-specific predictors for sepsis in polytrauma patients undergoing stand-alone definitive surgery. *Materials and Methods:* Our institutional trauma database, containing data from 3653 patients between 1996 and 2022, was filtered for polytrauma patients who underwent definitive surgery, were aged ≥16 years, and had an Injury Severity Score (ISS) ≥16. Injury and physiological parameters were documented upon admission, as well as on the first and second days thereafter. Surgical data were also recorded. All factors were evaluated for their association with sepsis development. *Results:* Among the 276 patients (71.1% male; mean age 45.0 years, 95% confidence interval 42.7–47.2 years; median ISS of 27, interquartile range, 20–34), the rate of sepsis was 13.8% (n = 38), with a higher incidence in males (16.7%; n = 33) than in females (6.8%; n = 5) (*p* = 0.026). Head and thorax injuries were more common and severe in septic males, with thorax injuries being predictive. Male patients who developed sepsis also had a higher ISS, an increased heart rate (HR), and lower pH at admission, with ISS and HR being predictive of sepsis. On the first day post-admission, septic males showed higher Base Excess and lower Prothrombin Time. Lactate levels were elevated on both the first and second days post-admission. Surgical predictors for males included less primary extremity surgeries and later secondary spine surgeries. *Conclusions:* Sepsis is more common in males undergoing stand-alone definitive surgery. Several factors, particularly acidosis and coagulopathy, are associated with this phenomenon. Fewer primary extremity surgeries and delayed secondary spine surgeries were also linked to a higher sepsis risk in males. These findings may help identify patients eligible for stand-alone definitive surgery and underscore the need for more data on female polytrauma patients.

## 1. Introduction

Polytrauma is common, particularly among young male patients [1,2] and remains a leading cause of death in young adults, thereby contributing to a substantial socioeconomic burden [3].

Triaging relies on injury and physiological parameters [4,5,6,7,8] to estimate the somatic stress induced by trauma. This stress, often referred to as the “first hit” [4,9], can trigger adverse events (AE), such as systemic inflammatory response (SIRS), as well as sepsis or mortality as a result of a compensatory anti-inflammatory response (CARS) [10].

The “second hit” resulting from surgical intervention [4,5] can be modified according to timing (early vs. delayed) and approach (damage control vs. definitive), if not directed by survival needs. This must balance early mobilization [11] with the potential stress caused by surgery [9,12].

Polytrauma patients with less severe injuries [4,12] and somatic stress often undergo isolated definitive orthopedic surgery without prior damage-control procedures [4,11].

AE, particularly sepsis—the leading cause of posttraumatic morbidity [13] and associated with increased costs and mortality [14]—should be minimized.

It has been suggested that males and females display different response patterns following polytrauma and should therefore be evaluated differently [1,3,15,16]. Male sex has been identified as a risk factor for sepsis following polytrauma [3,14,17,18,19,20], although age and injury severity must be accounted for [14,17,18,19,20]. The higher rate of sepsis in male polytrauma patients has largely been attributed to hormonal and immunological differences [2,3,15,16,17,18], specifically menopause [15], the estrous cycle [16], splenocytes [17], and cytokines [18].

The aim of this study was to identify sex-specific predictors for sepsis in polytrauma patients undergoing stand-alone definitive surgery, to provide time-dependent referencing, and to identify patients eligible for earlier surgical treatment.

## 2. Materials and Methods

We searched our institutional trauma database, which includes data from 3653 patients between 1996 and 2022, making this research a retrospective cohort study performed at a single institution (University Hospital Zurich).

After filtering for polytrauma patients who underwent definitive surgery (either early, on admission day, or delayed, thereafter), 1450 cases were identified. Considering only patients aged ≥ 16 years with an Injury Severity Score (ISS) ≥ 16 [21], 276 patients were included in the final analysis.

We assessed several injury factors (trauma type, accident type, ISS [22], and Abbreviated Injury Score (AIS) [21]) as well as physiological parameters [Glasgow Coma Scale, body temperature, body mass index (BMI), heart rate (HR), systolic blood pressure, mean arterial pressure, pH, Base Excess (BE), lactate, C-reactive protein, hemoglobin (HB), thrombocyte count, prothrombin time (PT), and leukocyte count]. These parameters were documented upon admission, and on the first and second days thereafter.

Regarding surgical parameters, the type and timing of procedures were recorded, along with the development of sepsis within 21 days of admission. Sepsis had to occur within the observational period of 21 days.

### 2.1. Definition of Sepsis

Sepsis was defined as the presence of a SIRS score of at least 2 in conjunction with a focus of infection [23,24]. An infection had to be diagnosed either by strong clinical criteria (hypotension, organ dysfunction, hypoperfusion) or by microbiological confirmation.

### 2.2. Laboratory Analysis

Laboratory analyses were performed using a standardized procedure. Point-of-Care-Testing (blood gas analysis) was specifically used for measurements of acid-base parameters. Analyses were performed at a single institution (Department of Clinical Chemistry) at the University Hospital Zurich.

### 2.3. Statistical Analysis

Data analysis was performed using SPSS 29.0 (IBM SPSS Statistics 29). For intergroup comparisons, a Chi-Square test, Mood’s median test, and a Mann-Whitney U-Test (due to missing normal distribution according to the Shapiro-Wilk test) were employed.

Binary logistic regression was used to identify predictive parameters for sepsis. If any parameters were identified upon admission, then the subsequent analogous analyses for the first and second days thereafter accounted for these confounding variables by including them in the regression model. The level of significance was set at *p* < 0.05.

### 2.4. Ethical Approval

Ethical approval for the study was granted by the Ethics Commission of the University Hospital of Zurich and the local government of Zurich upon database implementation (Nr. StV: 1-2008) and was later reapproved (BASEC 2021-00391). The study is congruent with good clinical practice, the Declaration of Helsinki, and the TRIPOD guidelines [25] for predictive modeling.

## 3. Results

In total, the 276 patients (71.1% male) presented with a mean age of 45.0 years (95% confidence interval, 42.7–47.2 years) and a median ISS of (interquartile range, 20–34) after sustaining almost exclusively blunt trauma (Table 1).

The rate of sepsis was 13.8% (n = 38), more common in males (16.7%; n = 33) than in females (6.8%; n = 5) (*p* = 0.026). In the female group, the septic focus comprised trachea-bronchial fluid (n = 2), bacteremia (n = 1), wound infection (n = 1), and urinary tract infection (n = 1). In the male group, the focus consisted of trachea-bronchial fluid (n = 20), bacteremia (n = 6), intravenous catheter (n = 3), cerebrospinal fluid infection (n = 2), and wound or urinary tract infection (n = 1, respectively).

There were several group differences and predictors (Table 2). Males suffered more work-related accidents and had a higher BMI, whereas females had more suicide cases, a higher rate of (and more severe) abdominal injuries, and a lower HB.

Regarding injury regions in septic males (Table 3), injuries to the head region were more common. Thoracic injuries were more common and severe in this group (especially AIS grade 3) and predictive of sepsis. Septic patients also had a higher ISS, a higher HR (borderline tachycardia), and lower pH, with the first two being predictive of sepsis.

On the first day after admission, septic males had higher BE and lower PT (Table 4). Lactate levels were higher on both the first and second days post-admission. Respective parameters for the female group are provided for comparison.

Regarding surgical parameters, predictive factors for males included less primary extremity surgery and a later secondary spine surgery (Table 5). Again, respective parameters for the female group are provided for comparison.

## 4. Discussion

This study investigates the sex-specific parameters influencing sepsis development in polytrauma patients undergoing stand-alone definitive surgery. It highlights that sepsis incidence is significantly higher in males despite comparable age and injury severity between sexes. Upon admission, males exhibited distinct injury patterns and physiological markers, with factors such as ISS, HR, and pH being associated with sepsis development. On the first day post-admission, septic males demonstrated lower BE and PT, alongside elevated lactate levels, which persisted on the second day. Fewer primary extremity surgeries and delayed secondary spine surgeries were linked to higher sepsis risk in males. Additional female data is required before sex-specific approaches to polytrauma patient management can be made.

Triaging of polytrauma patients typically relies on injury and physiological parameters [4,5,6,7,8]. Patients with less severe injuries [4,12] and lower somatic stress often undergo isolated definitive orthopedic surgery without damage-control procedures [4,11], although evidence supporting this approach is limited [12].

In this context, a “second hit” from surgical intervention [4,5,9,12] with a subsequent development of sepsis according to the CARS concept [10] must be avoided. This is particularly relevant for males, who are at higher risk of post-traumatic sepsis [3,14,17,18,19,20].

### 4.1. Comparative Literature

The current findings support previous reports of higher rates of sepsis in male polytrauma patients [3,14,17,18,19,20,26], especially when adjusted for age (younger patients being more at risk [2]) and injury severity [14,17,18,19,20], which were met in our study. It is important to note that the low number of septic cases in females represents a limitation that may mask potential differences and emphasizes the need for more data on female polytrauma patients undergoing definitive surgery.

The higher rates of sepsis in male polytrauma patients can be largely attributed to hormonal and immunological differences [2,3,15,16], although this cannot be confirmed directly by respective parameters (e.g., menopause [15], the estrous cycle [16], splenocytes [17], or cytokines [18]).

Regarding menopause, which usually occurs at 50 years of age in women [3], Trentzsch et al. [15] found higher rates of sepsis in male polytrauma patients compared to an age-matched female group, except for the age group of 55–64 years (i.e., postmenopausal females), demonstrating similar rates. This study is one of the few clinical studies suggesting a lower sepsis rate in association with estrogen (i.e., premenopausal women).

Mörs et al. compared males and females and, at a comparable age and ISS, reported higher values of the pro-inflammatory cytokine Interleukin-6 in males on the first and second days after admission, along with higher rates of sepsis in these polytrauma patients [18]. The females in this study had a mean age of 48.0 years.

In our study, males also had a higher BMI, though the absolute difference compared to females was small, and its clinical relevance should be questioned. A higher BMI is generally considered protective against septic complications [14,27] (provided it is not morbid obesity [27]), likely due to aromatase activity in fatty tissue [27]. This suggests that males had a higher rate of sepsis despite the protective factor of having a borderline overweight BMI.

### 4.2. Injury Profile

Male septic patients had a higher ISS. Injury severity is a main factor affecting outcomes after polytrauma [5,10,17,18,19], as it reflects the strain on the somatic system [4,9] and the compensatory response that may trigger sepsis [10]. Greater injury severity may also predispose patients to immobilization and the need for ventilation [5,10]. Although ISS differences according to sepsis development were only statistically significant in males, there was at least a tendency for the same observation in females, which should be considered.

Further analysis revealed that head injuries (particularly traumatic brain injury, TBI) and thoracic injuries in males are significant risk factors for sepsis [5,7,12,19,28]. This may partially be explained by reduced pulmonary ventilation [29] or complications arising from ventilation following severe head trauma. In contrast, females are more predisposed to abdominal injuries [2], although the association with their higher suicides rates versus fewer work accidents remains unclear and should be subject to further research, particularly as an injury to this region might be even more relevant if the woman is pregnant.

### 4.3. Physiological Parameters

At admission, there were no significant sex differences except for a generally lower HB level in females [2,15]. These values are physiological from a relative standpoint but should be further investigated to specifically identify their sex-specific relevance in the context of hemorrhage.

Laboratory differences observed in male septic cases at admission included a higher HR and lower pH/PT. A borderline tachycardia could serve as an early warning sign for sepsis [30], although its sensitivity decreases with age [30].

pH remains an established parameter for predicting sepsis after trauma [4,5,31,32], along with a lower BE on the first day and higher lactate levels on both the first and second days post-admission, expanding upon the relevance of a 24 h lactate parameter [6]. Although each parameter was elevated, they were only moderately so (below the threshold) [4,6,15,18,31,33,34] and responded to resuscitation [4]. Thus, a holistic, time-dependent approach with constant reassessment of persistent abnormalities is recommended [4,6,7], aligning with our practice of time-dependent evaluation [7,12,34]. Here, it must be mentioned that other established markers (procalcitonin [35] and cytokines [36]), which predict sepsis early and specifically but are also subject to heterogeneity in their predictiveness [35,36] (especially in light of sex differences, as indicated above) were not reported.

For PT, reduced levels were observed on the first day after admission, highlighting the importance of both acidosis [4,7,12,31,33] and coagulopathy [15,19,37], although cut-off values remain heterogenous despite comparable injury severities [15], emphasizing the need for an individual evaluation. Furthermore, PT alone is not particularly specific for coagulopathy, with viscoelastic coagulation being more reliable.

As the results are correlative rather than causative between parameters and sepsis, a sex-specific management strategy cannot be directly deducted. However, it appears that males should be monitored more closely by the investigated parameters and for sepsis development itself.

### 4.4. Surgical Factors

Fewer primary interventions and delayed secondary spine surgery were identified as factors that predisposed males to sepsis. There was at least a tendency for the same observation regarding a delayed secondary spine surgery in females, which should be considered.

This confirms Bone et al.’s principle [38] that early definitive treatment of long bones reduces pulmonary complications and intensive –care unit stays while facilitating early rehabilitation through mobilization [5,11]. Our findings suggest that time-dependent evaluation of parameters could improve decision-making by identifying patients who are eligible for earlier surgical treatment according to these criteria [7,11,12], where males might benefit from a slightly more restrictive surgery protocol to minimize their statistically higher risk of sepsis development.

### 4.5. Limitations

There are several limitations to note: Comorbidities, especially those potentially affecting acid-base-parameters, were not available in the data [14,39]. The results of this study describe factors associated with sepsis but have no causative implications.

Resuscitation protocols changed over the long study period [19]: They are now not only characterized by hypotensive/more restrictive resuscitation with less crystalloids and a (non-aggressive) administration of blood and plasma (related specifically to acid-base parameters via a dilution effect) but also by changes in the surgical strategy itself [6]. Additionally, antibiotic treatment has changed in light of multidrug-resistant microorganisms: While early administration still seems favorable, choosing the appropriate antibiotic based on patient history, microbiologic culture results, and community/epidemiologic considerations is crucial [40]. There may also be institutional (patient selection) bias, as our hospital is a level 1 trauma center frequently chosen for suspected/severe TBI cases. The low number of (female) septic cases limits the ability to detect sex-specific differences/conclusions (especially regarding ISS at admission for females in Table 3 or day of secondary spine surgery in Table 5) and highlights the need for further research on female polytrauma patients undergoing definitive surgery. In addition to socioeconomic factors, female polytrauma data is also inherently restricted by demographic trends, as males are more susceptible to traumatic injury internationally.

There is a need for multicenter studies and prospective data collection (especially of females) to validate these findings, enhance generalizability, and to address the limitations mentioned above.

Based on the findings of the current study in a specific cohort (undergoing definitive surgery), extending upon previous reports, there are sex-specific differences in sepsis development at a comparable age and injury severity, which may provide time-dependent referencing in the context of definitive surgery.

## 5. Conclusions

Sepsis is more common in males undergoing stand-alone definitive surgery. Several factors, particularly acidosis and coagulopathy, are associated with this phenomenon. From a surgical perspective, fewer primary extremity surgeries and delayed secondary spine surgeries were also linked to higher sepsis risk in males.

These findings may help better identify patients eligible for stand-alone definitive surgery and underscore the need for more data on female polytrauma patients undergoing definitive surgery.

## Figures and Tables

**Table 1:** Patient cohort characteristics upon admission.

Parameter at Admission	n	Value
Age	276	45.0 (42.7–47.2) ^1^
Sex (male)	276	71.7 (198) ^3^
Blunt trauma	276	97.1 (268) ^3^
Type of accident	276	13.0 (36) ^3^
-Work		
-Traffic		55.0 (152) ^3^
-Sports/Leisure		9.4 (26) ^3^
-At home		10.9 (30) ^3^
-Suicide		4.7 (13) ^3^
-Delinquency		0.7 (2) ^3^
-Other		6.2 (17) ^3^
ISS	276	27 (20–34) ^2^
AIS (0–5)		
-Head	276	26/12/8/24/14/16 (71/33/21/67/40/44) ^3^
-Face	276	66/7/19/8/1/0 (181/20/51/22/2/0) ^3^
-Thorax	276	35/3/5/40/14/3 (96/8/15/111/37/9) ^3^
-Abdomen	276	67/2/8/11/9/3 (185/4/21/31/26/9) ^3^
-Pelvis	276	68/1/10/17/4/0 (187/3/27/48/11/0) ^3^
-Spine	276	48/0/13/26/5/8 (133/0/37/71/14/21) ^3^
-Extremities	276	23/5/28/34/9/1 (63/14/79/93/25/2) ^3^
-Integument	276	63/26/10/1/0/0 (174/73/28/1/0/0) ^3^
GCS	270	14 (7–15) ^2^
Body temperature	200	35.9 (35.7–36.0) ^1^
BMI	197	24.2 (22.3–26.2) ^2^
HR	204	89 (87–92) ^1^
SBP	203	132 (129–135) ^1^
MAP	168	95 (92–97) ^1^
pH	197	7.34 (7.33–7.36) ^1^
BE	214	−2.36 (−2.80–−1.91) ^1^
Lactate	237	2.17 (2.00–2.33) ^1^
CRP	226	21.6 (14.5–28.7) ^1^
HB	246	12.1 (11.0–13.1) ^1^
TC	237	218 (207–229) ^1^
PT	230	87 (75–100) ^2^
LC	263	13.4 (12.8–14.1) ^1^

Age [years]; AIS, Abbreviated Injury Score; BE, Base Excess [mmol/L]; BMI, Body Mass Index [kg/m^2^]; CRP, C-reactive Protein [mg/L]; GCS, Glasgow Coma Scale; HR, Heart Rate [per minute]; HB, Hemoglobin [g/dL]; ISS, Injury Severity Score; Lactate [mmol/L]; LC, Leukocyte Count [G/L]; MAP, Mean Arterial Pressure [mmHg]; PT, Prothrombin Time [% of reagent]; SBP, Systolic Blood Pressure [mmHg]; Body Temperature [°C]; TC, Thrombocyte Count [G/L]. ^1^ Mean with 95% confidence interval; ^2^ Median with interquartile ranges; ^3^ Percentages with numbers.

**Table 2:** Parameters on admission day between females and males.

Parameter	n (Female vs. Male)	Values (Female vs. Male)	*p*-Value
Age	78 vs. 198	45.2 (40.7–49.7) vs. 44.9 (42.2–47.5) ^1^	0.936
Blunt trauma	78 vs. 198	98.7 (77) vs. 96.5 (191) ^3^	0.315
Type of accident	78 vs. 198		
-Work		0 vs. 18 (36) ^3^	<0.001
-Traffic		62.8 (49) vs. 52.0 (103) ^3^	0.104
-Sports/Leisure		9.0 (7) vs. 9.6 (19) ^3^	0.874
-At home		11.5 (9) vs. 10.6 (21) ^3^	0.823
-Suicide		13 (10) vs. 1 (3) ^3^	<0.001
-Delinquency		0 vs. 1.0 (2) ^3^	0.373
-Other		3.8 (3) vs. 7.1 (14) ^3^	0.316
ISS	78 vs. 198	27 (20–34) vs. 27 (20–35) ^2^	0.767
AIS (0–5)	78 vs. 198		
-Head		20/12/8/32/15/13 (16/9/6/25/12/10) vs. 28/12/8/21/14/17 (55/24/15/42/28/34) ^3^	0.467
-Face		67/9/17/7/0/0 (52/7/13/6/0/0) vs. 65/7/19/8/1/0 (129/13/38/16/2/0) ^3^	0.834
-Thorax		35/2/10/40/13/0 (27/2/8/31/10/0) vs. 35/3/4/40/14/4 (69/6/7/80/27/9) ^3^	0.142
-Abdomen		55/3/14/9/14/5/0 (43/2/11/7/11/4/0) vs. 72/1/5/12/8/2/0 (142/2/10/24/15/5/0) ^3^	0.02
-Pelvis		62/1/13/18/6/0 (48/1/10/14/5/0) vs. 70/2/9/17/3/0 (139/2/17/34/6/0) ^3^	0.508
-Spine		49/0/14/28/4/5 (38/0/11/22/3/4) vs. 48/0/13/25/5/9 (95/0/26/49/11/17) ^3^	0.825
-Extremities		24/6/28/30/12/0 (19/5/22/23/9/0) vs. 22/5/29/35/8/1 (44/9/57/70/16/2) ^3^	0.761
-Integument		63/28/8/1/0/0 (49/22/6/1/0/0) vs. 63/26/11/0/0/0 (125/51/22/0/0/0) ^3^	0.346
GCS	76 vs. 194	15 (11–15) vs. 14 (3–15) ^2^	0.235
Body temperature	55 vs. 145	35.7 (35.4–36.0) vs. 35.9 (35.7–36.1) ^1^	0.085
BMI	27 vs. 170	24.1 (22.0–26.1) vs. 25.4 (23.6–29.4) ^2^	0.009
HR	60 vs. 144	88 (83–93) vs. 90 (87–93) ^1^	0.653
SBP	60 vs. 143	128 (123–134) vs. 134 (130–138) ^1^	0.154
MAP	48 vs. 120	92 (88–97) vs. 96 (92–99) ^1^	0.175
pH	53 vs. 144	7.35 (7.33–7.37) vs. 7.34 (7.33–7.36) ^1^	0.372
BE	58 vs. 156	−2.19 (−2.89–−1.50) vs. −2.42 (−2.98–−1.85) ^1^	0.813
Lactate	69 vs. 168	2.09 (1.84–2.35) vs. 2.20 (2.00–2.41) ^1^	0.929
CRP	61 vs. 165	15.2 (2.5–27.9) vs. 24.0 (15.4–32.6) ^1^	0.895
HB	69 vs. 177	12.2 (11.0–13.4) vs. 11.3 (10.4–12.1) ^1^	<0.001
TC	63 vs. 174	221 (205–237) vs. 217 (203–231) ^1^	0.345
PT	61 vs. 169	90 (75–100) vs. 87 (75–99) ^2^	0.214
LC	76 vs. 187	12.8 (11.6–14.0) vs. 13.7 (12.9–14.5) ^1^	0.339

AIS, Abbreviated Injury Score; BE, Base Excess [mmol/L]; BMI, Body Mass Index [kg/m^2^]; CRP, C-reactive Protein [mg/L]; GCS, Glasgow Coma Scale; HR, Heart Rate [beats per minute]; HB, Hemoglobin [g/dL]; ISS, Injury Severity Score; Lactate [mmol/L]; LC, Leukocyte Count [G/L]; MAP, Mean Arterial Pressure [mmHg]; PT, Prothrombin Time [% of reagent]; SBP, Systolic Blood Pressure [mmHg]; Body Temperature [°C]; TC, Thrombocyte Count [G/L]. ^1^ Mean with 95% confidence interval; ^2^ Median with interquartile ranges; ^3^ Percentages with numbers.

**Table 3:** Parameters on admission day according to sepsis development.

Males						
Parameter	n	Value-No Sepsis-	n	Value-Sepsis-	*p*-Value	Sepsis (BLR)
AIS						
-Head	165	26/10/8/25/15/16/0 (43/17/14/41/24/26/0) ^3^	33	37/21/3/3/12/24/0 (12/7/1/1/4/8/0) ^3^	0.031	0.929
-Thorax	165	38/4/4/36/15/3/0 (63/6/7/60/24/5/0) ^3^	33	18/0/0/61/9/12/0 (6/0/0/20/3/4/0) ^3^	0.009	0.018
ISS	165	26 (20–34) ^2^	33	35 (26–40) ^2^	<0.001	<0.001
HR	120	88 (85–91) ^1^	24	99 (89–109) ^1^	0.022	0.036
pH	120	7.35 (7.33–7.36) ^1^	24	7.31 (7.27–7.35) ^1^	0.039	0.162
**Females**						
**Parameter**	**n**	**Value-No Sepsis-**	**n**	**Value-Sepsis-**	***p*-value**	**Sepsis (BLR)**
AIS						
-Head	73	21/11/8/33/16/11/0 (15/8/6/24/12/8/0) ^3^	5	20/20/0/20/0/40/0 (1/1/0/1/0/2/0) ^3^	0.430	0.450
-Thorax	73	36/3/9/38/14/0/0 (26/2/7/28/10/0/0) ^3^	5	20/0/20/60/0/0/0 (1/0/1/3/0/0/0) ^3^	0.699	0.747
ISS	73	27 (20–34) ^2^	5	35 (26–42) ^2^	0.103	0.126
HR	57	88 (83–93) ^1^	24	92 (48–137) ^1^	0.653	1
pH	50	7.35 (7.33–7.37) ^1^	3	7.36 (7.18–7.54) ^1^	1	1

AIS, Abbreviated Injury Score; BLR, Binary Logistic Regression; HR, Heart Rate [beats per minute]; ISS, Injury Severity Score. ^1^ Mean with 95% confidence interval; ^2^ Median with interquartile ranges; ^3^ Percentages with numbers.

**Table 4:** Parameters on the first (1) and second (2) days post-admission according to sepsis development.

Males						
Parameter	n	Value -No Sepsis-	n	Value -Sepsis-	*p*-Value	Sepsis (BLR)
BE1	97	1.25 (0.79–1.72) ^1^	22	1.44 (−1.52–4.39) ^1^	0.035	0.636
Lactate1	123	1.10 (0.99–1.21) ^1^	27	1.56 (1.31–1.82) ^1^	<0.001	0.306
Lactate2	73	1.06 (0.90–1.22) ^1^	19	1.37 (1.15–1.59) ^1^	0.002	0.407
PT1	150	91 (78–100) ^2^	22	82 (68–99) ^2^	0.035	0.124
**Females**						
**Parameter**	**n**	**Value** **-No Sepsis-**	**n**	**Value** **-Sepsis-**	***p*-Value**	**Sepsis (BLR)**
BE1	52	−0.42 (−1.30–0.46) ^1^	3	−0.60 (−3.22–2.02) ^1^	0.635	0.920
Lactate1	59	1.05 (0.86–1.24) ^1^	4	1.25 (0.27–2.23) ^1^	0.290	0.974
Lactate2	37	1.11 (0.89–1.32) ^1^	2	1.15 (0.20–2.18) ^1^	0.734	0.680
PT1	49	94 (81–100) ^2^	3	85 (84–86) ^2^	0.552	0.973

BE, Base Excess [mmol/L]; PT, Prothrombin Time [% of reagent]; Lactate [mmol/L]; BLR, Binary Logistic Regression. ^1^ Mean with 95% confidence interval; ^2^ Median with interquartile ranges.

**Table 5:** Surgical parameters predicting sepsis.

	Males		Females	
	Percentage (n)	*p*-Value	Percentage (n)	*p*-Value
Primary extremity surgery	29% (n = 58)	0.039 (r = −1.064)	32% (n = 25)	0.242
Day of secondary spine surgery	Total: 27% (n = 53) 1: 7% (n = 14) 2: 6% (n = 12) 3 and after: 14% (n = 27)	0.02 (r = 0.226)	Total:32% (n = 25) 1: 9% (n = 7) 2: 10% (n = 8) 3 and after: 13% (n = 10)	0.085

*p*-values are presented with the exponentiation coefficient if significant. If procedures are reported by respective day, their sum is described first.

## Data Availability

Dataset available on request from the authors.
